# Home Department

**Published:** 1886-06

**Authors:** 


### HOME DEPARTMENT.

**Bread.**—Dogs fed by Magendie on flour bread died in forty days ; other dogs fed on bread made of whole wheat, meal or flour flourished and throve. The three-fourths impoverishment of the mineral ingredients proved fatal to the first. Why should not makind suffer in some manner for living on impoverished food ?

**Wheat.**—The history of the Roman Empire in the time of Julius Cæsar shows that wheat as an article of food, with the out-door life, is capable of producing and sustaining the highest type of physical manhood the world has ever seen. The empire was built up and sustained by soldiers, whose chief article of food was wheat.

**Graham vs. Fine Flour.**—The following table is furnished by a Mr. Johnson, in *Blackwood's Magazine.*

| In 1,000 lbs. | Whole Wheat. | Fine Flour. |
|---|---|---|
| Muscular matter.......... | 156 lbs. | 130 lbs. |
| Bone and saline matter.... | 170 " | 60 " |
| Fatty matter............. | 28 " | 20 " |
| | 354 lbs. | 210 lbs. |

It will be seen by this how much is lost by eating fine flour bread with most of the real life-giving principle deducted.

**The Health of Pork.**—Dr. James C. Jackson once made a statement which he said he based on information derived from the pork dealers of Cincinnati, Ohio, that "ninety-five hogs in one-hundred have *ulcers* on their livers from the size of an ounce bullet to a hen's egg," and yet thousands eat the hog, liver and all.

**Oatmeal Crisp.**—Scald the oatmeal with boiling water, stirring with a spoon, and make a pretty stiff dough ; knead well together, dust the moulding-board with a little Graham flour, and roll to a thickness of nearly half an inch. Then cut into small cakes and bake in an oven fifteen minutes or more until they are dry and hard, but only slightly browned. Watch closely that they do not scorch in baking. In rolling out this dough it cracks badly near the edges ; after using the cake cutter gather up the ragged pieces, knead them well together, and roll again.

**Mush Rolls.**—Take any cold mush made of corn meal, graham or rye flour, oat meal, samp or farina, and knead into it enough graham flour to form rather a soft dough; just stiff enough to handle with plenty of flour. If too much or too little flour is worked in, the bread will not be good. Make into rolls three or four inches long and nearly an inch thick; then bake in a hot oven thirty or forty minutes. This bread is best eaten warm.

**To Quench Thirst.**—A feverish thirst that cannot be quenched by water may be allayed thus: Throw a slice of bread upon burning coals, and when it is aflame put it into a tumbler of water and drink the liquid. This remedy has been tested and proved excellent.

**Stale Bread.**—There are two excellent ways of disposing of very stale bread, providing it is neither sour nor mouldy. If it is in sundry small pieces and is soaked in water or milk for a short time, it will with good management make excellent pudding. If in pieces large enough to make slices, cut it first, then dip the slices in water, and in a few minutes toast as fresh bread. If the secret of the staleness is concealed, this toast when served buttered will be preferred to that made with fresh bread.

**Chicory.**—Chicory is a root somewhat resembling parsnip, which, when cut in pieces roasted and ground, resembles coffee. It is extensively imported from Germany, Holland and Belgium, and is also grown in Lincolnshire and elsewhere in England, subject to an import duty, and an excise duty upon home productions. A small quantity added to coffee is an improvement, but is more usually added in much greater proportion, especially when sold "mixed." Chicory itself is not always genuine but often made up or mixed with roasted corn, various refuses, ground mahogany, and other woods, damaged flour and biscuit, acorns, horse-chestnuts, baked horses' livers, Venetian red, &c. The only security is to buy it separately, and to test it by dusting some on the surface of a basin of water. The chicory will speedily sink to the bottom and the other matters float at the top.

**Caseine.**—Caseine is in its properties distinct from albumen and fibrine. It is held in solution in milk by an alkali, and may be heated to boiling point without coagulation. Diluted acids which do not coagulate albumen, easily seperate the caseine from milk; it is easily coagulated in the cold by diluted acetic acid, and separates in the form of a jelly. The analysis of caseine has proved that this body also contains the same elements and in the same proportion as albumen and fibrine, with the exception that it contains a little less sulphur.

**Cheese.**—The value of cheese as food consists in the great proportion of caseine it contains. Caseine coagulates by the action of acids only.

